# Vesicular Trafficking, a Mechanism Controlled by Cascade Activation of Rab Proteins: Focus on Rab27

**DOI:** 10.3390/biology12121530

**Published:** 2023-12-15

**Authors:** Camille Menaceur, Océane Dusailly, Fabien Gosselet, Laurence Fenart, Julien Saint-Pol

**Affiliations:** Univ. Artois, UR 2465, Blood-Brain Barrier Laboratory (LBHE), F-62300 Lens, France; camille.menaceur@univ-artois.fr (C.M.); oceane.dusailly@univ-artois.fr (O.D.); fabien.gosselet@univ-artois.fr (F.G.); laurence.tilloy@univ-artois.fr (L.F.)

**Keywords:** RabGTPase, vesicular trafficking, extracellular vesicles, Rab27a, Rab27b

## Abstract

**Simple Summary:**

Vesicle trafficking is governed by the careful regulation of RabGTPase activation/inactivation, which promotes vesicle formation from a donor membrane, motility, docking/tethering and fusion with a recipient membrane. This review highlights the main Rab proteins involved in these processes under physiological conditions, and focuses on the pathophysiological role of the two Rab27 isoforms. This review presents the interest in studying Rab proteins and their effectors in the regulation of vesicular trafficking, and opens the interest of considering them in pathological conditions as outcomes for targeted therapeutical approaches.

**Abstract:**

Vesicular trafficking is essential for the cell to internalize useful proteins and soluble substances, for cell signaling or for the degradation of pathogenic elements such as bacteria or viruses. This vesicular trafficking also enables the cell to engage in secretory processes for the elimination of waste products or for the emission of intercellular communication vectors such as cytokines, chemokines and extracellular vesicles. Ras-related proteins (Rab) and their effector(s) are of crucial importance in all of these processes, and mutations/alterations to them have serious pathophysiological consequences. This review presents a non-exhaustive overview of the role of the major Rab involved in vesicular trafficking, with particular emphasis on their involvement in the biogenesis and secretion of extracellular vesicles, and on the role of Rab27 in various pathophysiological processes. Therefore, Rab and their effector(s) are central therapeutic targets, given their involvement in vesicular trafficking and their importance for cell physiology.

## 1. Introduction

**Figure 1:**
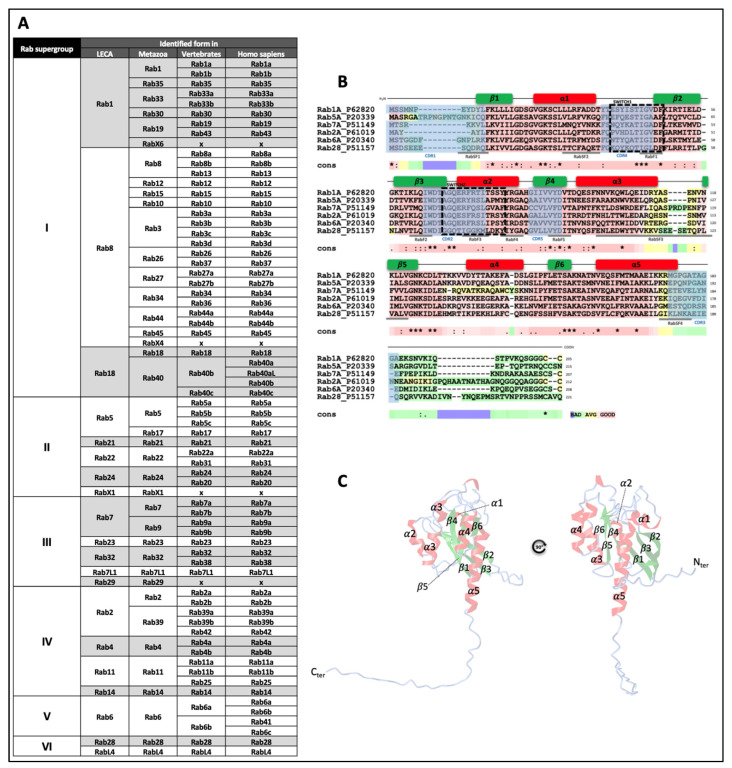
Rab expressions throughout evolution, structure and cycle of Rab activation/inactivation. (**A**) Expressions of the six superfamilies of Rab-GTPases from the latest established common ancestor (LECA) to humans. Crosses reflect the loss of some Rab during evolution. (**B**) Sequence alignment of selected Rab and delimitation of the conserved 5 α-helices and 6 β-sheets, switch domains, Rab family (RabF1-5) and subfamily (RabSF1-4) motifs, complementary determining regions (CDR1-5). Stars (*) represent the conserved amino acids, colons (:) for amino acids residues with similar physicochemical properties, points (.) for semi-conserved substitutions [9]. (**C**) AlphaFold structure of Rab27a highlighting the conserved α-helices and β-sheets [10,11,12].

**Figure 2:**
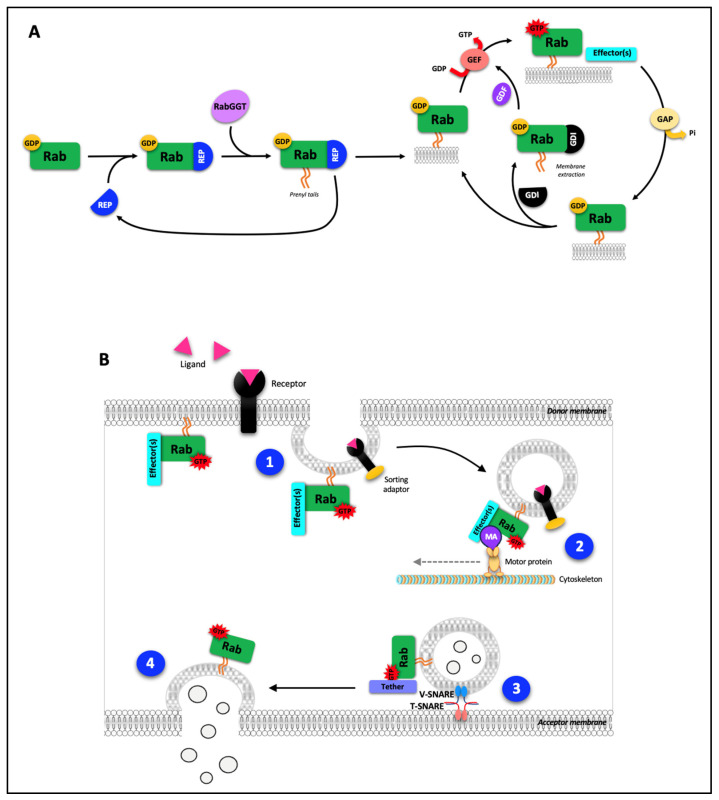
Cycle of Rab activation/inactivation (**A**) and main functions of Rab in vesicular trafficking (**B**). 1: Vesicle formation from a donor membrane; 2: vesicle motility and delivery to organelles/target membrane; 3: vesicle tethering and docking; 4: vesicle fusion with acceptor membrane and/or release of vesicle contents. GAP: GTPase activating protein; GDF: GDI displacement factor; GDI: guanine nucleotide dissociation inhibitor; GDP: guanine diphosphate; GEF: guanine nucleotide exchange factor; GTP: guanine triphosphate, MA: motor adaptor; RabGGT: Rab geranylgeranyl transferase; REP: Rab escort protein.

Since their discovery in the late 1980s [1], numerous studies have highlighted the involvement of Rab (Ras-related proteins), which belong to the Ras GTPase superfamily, in many different cellular processes ranging from endocytosis to intracellular vesicles formation, movement, tethering and fusion [2,3,4,5]. Rab are also involved in the genesis of extracellular vesicles. Rab proteins are well conserved through evolution, highlighting their major roles for cell functions. From 20 Rab from the latest established common ancestor in 6 Rab supergroups, the number of Rab is increasing throughout evolution, following the complexity of organisms: 39 in Metazoa, 62 in Vertebrates and 66 in humans (Figure 1A). In terms of structure, Rab exhibit conserved amino acid sequences that reflect a common structure, with five α-helices and six β-sheets and two switch domains (Figure 1B,C). Some domains, referred to as Rab family and Rab subfamily motifs (RabF1-4 and RabSF1-5 respectively), reflect Rab functions and interactions with Rab-interacting proteins such as GTPase activating protein (GAP), guanine nucleotide exchange factor (GEF) and guanine nucleotide dissociation inhibitor (GDI) [6,7]. Moreover, complementary determining regions (CDR1-5) are domains involved in the specificity of Rab effectors recruitment [8]. Despite their different cellular locations, these proteins function according to the same cycle of activation and inactivation (Figure 2A). The free inactive form of the Rab protein, coupled to guanine diphosphate (GDP), is first taken up by a chaperone protein, Rab escort protein (REP), and then acquires a prenyl anchor through the action of Rab geranylgeranyl transferase (RabGGT), enabling it to bind to the membrane of the target organelle.

The transition from the inactive to the active form of Rab proteins occurs (i) under the control of GEF, which catalyzes the reaction of GDP to guanine triphosphate (GTP) and (ii) by association with effector proteins. Conversely, the transition from the active to the inactive state occurs through the action of GAP. Rab are then released from the membrane by association with GDI or can resume an inactivation/activation cycle [13]. Rab also have different post-translational modification sites for phosphorylation, adenynylation and prenylation (reviewed in [14]); however, phosphorylation of Rab can modulate their activation/inactivation cycle by modifying the affinity and function of GAP, PEF and effectors [7,15,16], as well as their membrane targeting as observed for Rab4 during mitosis [17]. Rab phosphorylation is also able to modulate their functions and their abundance in the cytoplasm [7,18,19,20,21].

Rab proteins are involved at different levels of vesicular traffic. From a donor membrane, whether plasma membrane or organelle membrane, Rab promote the formation of vesicles and ensure their motility in association with motor elements of the cytoskeleton, notably microtubules (Figure 2B 1, 2). Rab also enable vesicles to be tethered to a recipient membrane, organelle or plasma, through the intervention of a tether effector and SNAREs proteins. This tethering leads to fusion of the vesicle concerned with the recipient membrane and the release of its contents into the intra- or extracellular recipient compartment (Figure 2B 3, 4).

This short review presents a non-exhaustive overview of the involvement of the main Rab proteins during the different stages of vesicular formation and tethering, trafficking, as well as their activation/inactivation cascade, which is essential for endosome routing, but also in exocytosis pathways (Table 1).

## 2. Involvement of Rab in Endosome Routing

**Figure 3:**
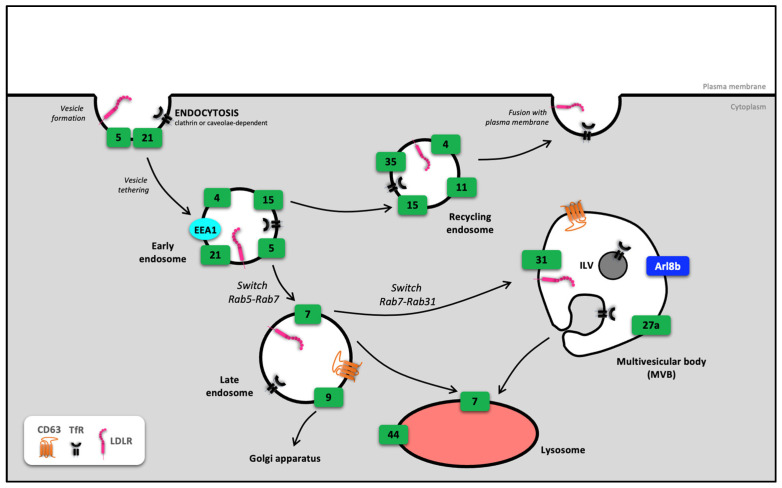
Endosomal routing by Rab proteins. The cascade of inactivation of one Rab/activation of another Rab directs the fate of the early endosome towards (i) a recycling pathway at the plasma membrane, (ii) an endolysosomal degradation pathway or (iii) the multivesicular body. For greater clarity, the Rab involved in this routing are symbolized by green rectangles. Arl8b: ADP-ribosylation factor-like protein 8B; EEA1: early endosome antigen 1; ILV: Iintraluminal vesicle, LDLR: low-density lipoprotein receptor; TfR: transferrin receptor.

Rab proteins are being extensively studied for their involvement in the endosomal machinery. This enables proteins to be sorted out for recycling and those to be directed towards the lysosomal degradation pathway. Once internalized, the membrane invagination that becomes an early endosome after splitting with the plasma membrane will mature into a recycling endosome or a late endosome, depending on its content. In recycling endosomes, the proteins to be recycled are transported to the cell membrane. This is the case for the low-density lipoprotein receptor (LDLR), transferrin receptor (TfR) [22,23,24]. The elements contained in the late endosomes will either be directed towards the trans-Golgi network, degraded or, as observed in polarized cells, brought unchanged from one pole of the cell to the other by the process of transcytosis [22]. Most of the cargo will be transported to the lysosome for degradation. During this endolysosomal process, a progressive acidification of the compartments is observed, enabling the activation of proteolytic enzymes linked to degradation. The role of Rab proteins at this level is to orientate or sort the endosomes according to their cellular fate (Figure 3). For example, activation of Rab4, Rab11, Rab15 and Rab35 leads the early endosome to become a recycling endosome, while activation of Rab7 determines its fate as a late endosome [25].

### 2.1. From Early Endosome to Recycling Endosome

The early endosome, generally associated with the protein marker early endosome antigen 1 (EEA1), contains various Rab proteins in active and inactive forms, such as Rab4, Rab 5, Rab15 and Rab21. Rab5 is activated in early endosomes and, like the EEA1 protein (one of its effectors), appears to be a marker of early endosomes [4]. Rab5 activation is also known to regulate clathrin- and caveolin-1-mediated endocytosis pathways [26]. However, a recent study in cortical neurons has refined this process. In fact, Rab21, and not Rab5, regulates caveolin-1-dependent endocytosis involved in the pruning of immature neurites, demonstrating the existence of two distinct populations of early endosomes, Rab5^+^ or Rab21^+^. Therefore, Rab5 would be preferentially associated with the clathrin-dependent endocytosis pathway [27].

Proteins endocytosed and transported by early endosomes may follow a recycling pathway to the plasma membrane, depending on the activation of other Rab. Indeed, the two Rab4 isoforms, a and b, have been described as being involved, once activated, in the recycling to the plasma membrane of various proteins once activated by its effectors such as CD2AP, dynein intermediate light chain-1 (dynein LIC-1) and syntaxin-4 [28,29,30]. In addition, activation of Rab15 by its effector, Rab15 effector protein (REP15), allows recycling of TfR to the plasma membrane according to research carried out on HeLa cells [31]. Rab11 has been described as a marker of recycling endosomes [32], and is notably involved in the recycling of myosin Vb to the plasma membrane following activation by its effector, Rab11 family interacting protein 2 (Rab11-FIP2) [33]. Compared with Rab4, which regulates rapid recycling, Rab11 is responsible for a slower recycling pathway [19]. Finally, the role of Rab35 in recycling is not precisely known, but it remains essential for the final stages of cytokinesis by controlling the subcellular localization of septin and phosphatidylinositol 4,5-bis phosphate (PIP2) during cell division in humans and drosophila [34].

### 2.2. From the Early Endosome to the Late Endosome

During the transition from the early endosome to the late endosome, a process of Rab conversion or switch is observed, with a depletion of Rab5 and an enrichment of Rab7. This mechanism is made possible by Rab5 recruiting the Rab7 GEF complex, thereby activating Rab7; Rab7 recruits the Rab5 GAP complex, thereby inactivating Rab5 [35]. One study showed that inhibition of Rab5 expression by more than 50% in liver cells had a considerable impact on the endosomal system, highlighting its essential role in endosomal maturation [36]. In late endosomes, Rab9 in its activated form is often associated with the recycling of cargo to the Golgi apparatus [37]. However, Rab9 is also involved in regulating the size and stability of the late endosome following interaction with its effector, the tail-interacting protein of 47 kDa (TIP47) [38].

### 2.3. Fate of the Late Endosome

Although Rab7 is the major player in the early endosome–late endosome transition, it also plays a major role in the fusion of late endosomes and subsequent multivesicular bodies (MVB) with the lysosome through mechanisms involving a tether complex formed by Rab7, N-ethylmaleimide sensitive factor (NSF) and soluble NSF attachment proteins (SNAPs), and SNAp receptor (SNAREs) proteins for the fusion between MVB and lysosome, including vesicle-associated membrane proteins 7 and 8 (VAMP7-8) and syntaxins 7 and 8 [39,40]. Rab7 deficiency disrupts this late endosome–lysosome transition, but also autolysosome formation and autophagosome maturation [41]. Although present in lysosomes, the role of Rab44 in lysosomal functions remains unclear, especially as its subcellular localization appears to be influenced by intracellular Ca^2+^ levels [13]. However, it has been reported that Rab44 plays a role in lysosomal exocytosis in mast cells, but this mechanism depends on the Rab44 isoform involved [42].

At the level of the late endosome, the Rab cascade that leads to the inactivation of Rab7 determines its fate towards the MVB. Activation of Rab31 leads to the recruitment of a GTPase and the TBC1 domain family member 2B (TBC1D2B) protein, which inactivates Rab7 and prevents MVB fusion with the lysosome [43]. Another GTPase is recruited to the MVB, the ADP-ribosylation factor-like protein 8B (Arl8b), which plays a role in the secretion of intraluminal vesicles (ILV, see Section 2.1).

From endocytosis onwards, the switch from one Rab to another, i.e., the activation/inactivation of Rab and their recruitment to organelles, determines the fate of the endosome towards recycling, degradation or maturation pathways, opening up other cellular processes such as biogenesis and extracellular vesicles secretion.

## 3. Role of Rab Proteins in the Biogenesis and Secretion of Extracellular Vesicles

**Figure 4:**
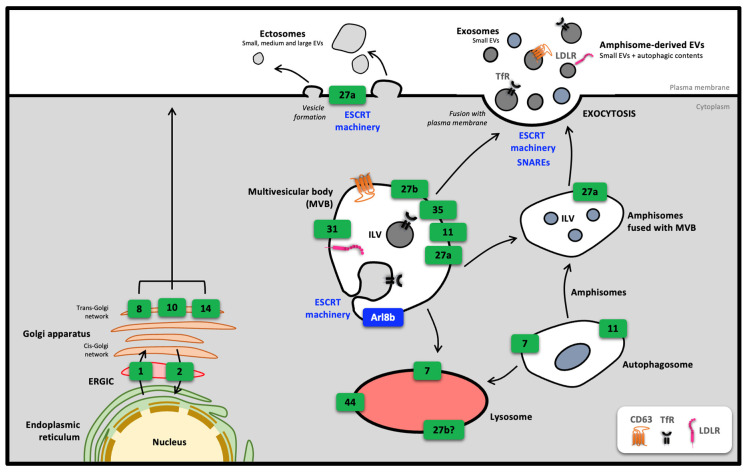
Involvement of Rab in cell secretion mechanisms. Rab proteins participate in various secretory pathways, as shown here, originating from the Golgi apparatus, or from the multivesicular body (MVB) and its potential fusion with amphisomes for the biogenesis and secretion of extracellular vesicles (EVs). EVs can also arise from plasma membrane budding under the action of Rab27a and the ESCRT machinery. For clarity, Rab proteins are symbolized by green rectangles. Arl8b: ADP-ribosylation factor-like protein 8B; ERGIC: endoplasmic reticulum–Golgi intermediate compartment; ESCRT: endosomal sorting complexes required for transport; EVs: extracellular vesicles; ILV: intraluminal vesicles; LDLR: low-density lipoprotein receptor; SNAREs: SNAp receptors; TfR: transferrin receptor.

In addition to their role in endosome routing, Rab proteins are also involved in various cellular secretory processes. Examples include the role of Rab8, Rab10 and Rab14 in addressing vesicles from the trans-Golgi network to the plasma membrane (Figure 4). In this section, the importance of Rabs in the biogenesis and secretion of extracellular vesicles (EVs) will be discussed.

### 3.1. Biogenesis and Secretion of Extracellular Vesicles

Over the last two decades, there has been growing interest in the involvement of EVs in cellular communication. Initially described as vectors for the elimination of cellular waste, EVs are now being studied for their ability to exchange messenger compounds (proteins, nucleic acids of the microRNA (miRNA) type, soluble peptides and/or enzymes, etc.) between cells over short or long distances. This mode of cell–cell communication is important in the pathophysiological regulation of recipient cells, and as soon as the protein profile of EVs varies in pathological conditions, these EVs are considered as potential biomarkers [44,45]. EVs are classified into three main categories according to their size: small EVs with a diameter of between 40 and 150 nm, including vesicles known as “exosomes” and “small ectosomes”; medium-sized EVs with a diameter of less than 500 nm; and large EVs with a diameter of more than 500 nm [46,47,48]. Of all the EVs, small EVs have been the most widely described, particularly for their involvement in cell–cell communication mechanisms. Small EVs can originate (i) by budding from the plasma membrane [43] to the external environment or (ii) by endosomal biogenesis, i.e., they originate in MVB in the form of ILV.

Currently, around ten Rab proteins have been identified as being involved in the various stages of EVs biogenesis/secretion. It has also been shown that inhibition by RNA interference of certain Rab proteins such as Rab5 or Rab27 has consequences for the secretion of small EVs [49]. During the ILV formation stage within MVB, several biogenesis mechanisms have been described. The first, and best known, is the secretion machinery-dependent biogenesis pathway ESCRT (endosomal sorting complexes required for transport), a protein cascade allowing invagination and scission of MVB membranes towards their lumen [45]. MVB naturally fuse with the lysosome, but the latter is diverted from its function in the degradation pathway by the inactivation of Rab7 by Rab31 and taken to the periphery of the cell to fuse with the PM, and thus release in the form of small EVs the ILV contained in its lumen. Arl8b is recruited to MVB, allowing centripetal movement of MVB towards the cell periphery [43,50]. A recent study highlighted an ESCRT-independent biogenesis pathway involving Rab31. Rab31 promotes the involvement of flotillins in MVB lipid rafts, resulting in preferential targeting of the epidermal growth factor receptor (EGFR) in CD63^+^ ILV [43]. Rab11 appears to be involved in MVB docking to the PM prior to calcium-dependent fusion [51,52]. It has also been reported that reduced Rab11 activity in K562 myeloid leukemia cells affects exosome secretion [53] but was not found in HeLa cells [49], suggesting a cell type-dependent involvement of Rab11. Rab35 has also been reported to be involved in exosome secretion in hepatocellular carcinoma [54] and in the fusion steps of MVB to PM [51].

A recent study looked at the molecular profile of MVB fusing with PM. Using a microscopic approach, it was shown that MVB fusing to the PM are positive only for Rab27, and not for other GTPases such as Rab7a and Arl8b. During its movement towards the PM, MVB are depleted of Arl8b, which is replaced by Rab27a, making fusion with the PM possible [55]. Therefore, CD63^+^ MVB fusing with the PM would be decorated essentially with Rab27a and b proteins, which appears to be essential in EVs biogenesis.

### 3.2. Importance of Rab27 in Pathophysiological Processes

**Figure 5:**
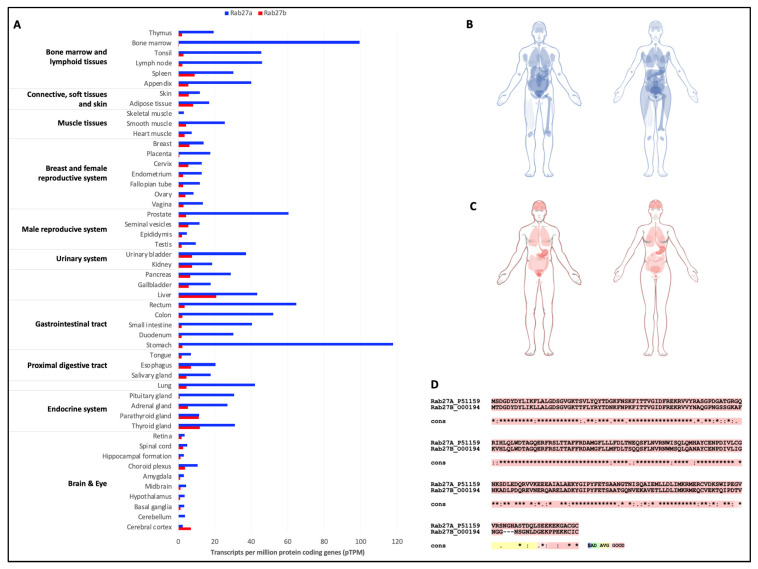
Expressions of Rab27 isoforms in the body and comparison of their protein sequences. Comparative analysis of Rab27a and Rab27b expressions in tissues (**A**), and graphical view of the expressions in the male and female body for Rab27a (**B**) and Rab27b (**C**). Data extracted from Human Protein Atlas [60]. (**D**) Sequence alignment of Rab27a and Rab27b proteins. Stars (*) represent the conserved amino acids; colons (:) represent amino acids residues with similar physicochemical properties; points (.) represent semi-conserved substitutions [9].

Rab27 isoforms belong to the Rab superfamily I, with a structure close to Rab8 (Figure 1A,C). The two Rab27 isoforms, Rab27a and Rab27b, are expressed in all tissues, but it is important to note that Rab27a is the main isoform expressed in the body and is particularly enriched in bone marrow, lymphoid tissues, prostate, stomach and the overall gastrointestinal tract. Despite a lower expression than Rab27a, Rab27b expression is high in the liver and in thyroid glands (Figure 5A–C). Rab27a and Rab27b are expressed by distinct genes, and exhibit around 70% gene sequence homology. Protein sequence alignment exposes a very high homology of amino acids between both isoforms, differing in a small region of the C-terminal region (Figure 5D). Both proteins are present in MVB, but also in PM. Rab27b is preferentially located in the trans-Golgi network, while Rab27a is often associated with CD63^+^ compartments (25%, compared with 10% for Rab27b) [49,55]. The role of the Rab27 protein has been extensively documented in melanocytes, where it participates in the migration of melanosomes towards the PM under the control of its two effectors, melanophilin (MLPH) and synaptotagmin-like protein 2-a (Slp2a) [4]. Rab27 and effectors, and particularly Rab27a, have been described to be of importance for secretion pathways as described for secretory granule exocytosis [56], and in docking/tethering of intracellular vesicles with acceptor membranes [57].

Inhibiting the protein expressions of the two Rab27 homologs has led to a better understanding of their role in the biogenesis of EVs of different sizes. RNA interference inhibition of the two Rab27 homologs reduces the secretion of small EVs by disrupting SNAREs (soluble N-ethylmaleimide-sensitive-factor attachment protein receptors) and the actin-dependent exocytosis system. However, inhibition of these proteins had no impact on the composition of the few EVs secreted by shRab27a and shRab27b cells. Therefore, Rab27a and Rab27b are thought to play a role in the docking stages of MVB to the PM, and inhibiting their respective effectors, synaptotagmin-like protein 4 (SYP4) and exophilin protein 5 (EXPH5), induces the same cellular responses. More specifically, Rab27b is involved in MVB motility towards the cell periphery, and both isoforms are thought to be important for the fusion step [49]. Rab27s are also involved in the amphisome biogenesis pathway. They are involved in the fusion of the autophagosome with the PM, releasing small extracellular vesicles from the amphisomes [58]. Rab27a is also involved in the formation of microvesicles (medium-sized EVs) and ectosomes (small EVs) in the PM by reorganizing the actin cytoskeleton, a mechanism that is essential for vesicular trafficking and plasma membrane deformation. Rab27b has been described to be involved in lysosomal exocytosis in oligodendrocytes [59].

Given the major involvement of Rab27 in vesicular trafficking, it is not surprising that some studies have focused on this protein from a pathological context. It has been described that, in a context of ovarian cancer that is resistant to cisplatin (an anti-cancer treatment), an overexpression of Rab27a is observed in association with an increase in the EVs biogenesis machinery and a disturbance in lysosomal function. Inhibition of Rab27a expression by RNA interference restores lysosomal function in treatment-resistant cells, with a morphology approaching that of cisplatin-sensitive cells [58]. Although Rab27s appear to be involved in cancerous processes, their pathological impact has been more extensively documented in the context of melanogenesis. Indeed, the *Rab27a* mutation was the first Rab mutation to be associated with an inherited disease characterized by neurological disorders, a pigmentation defect (partial albinism, silver sheen in the hair) and an immune deficiency, human Griscelli syndrome type 2 [61]. Loss of function of Rab27a or mutation of its effectors Mlph and Slp2a in melanocytes results in a defect in the formation of the Rab27a-Mlph-Myosin Va complex, which is responsible for actin-dependent anterograde transport of melanosomes to the plasma membrane and accumulation of melanosomes in perinuclear spaces [61]. Rab27b is also capable of interacting with Mlph and Slp2a, but is not expressed by melanocytes. In contrast, an overexpression of Rab27b in Rab27a-deficient melanocytes restores anterograde transport of melanosomes to the PM [62].

In addition to Griscelli syndrome type 2, mutations in Rab27a are also responsible for serious diseases based on a deficiency in lytic granule exocytosis in T lymphocytes [62]. However, no human diseases have been associated with Rab27b mutations. The use of Rab27a- and/or b-deficient mouse models has demonstrated an impact on different cell types, such as pancreatic cells or immune cells, indicating that a Rab27 protein deficiency could be involved in various diseases [63]. A recent study carried out a mathematical analysis of the involvement of Rab27 proteins in solid tumors. A significant relationship exists between the expression of Rab27, particularly Rab27b, and poor survival following the diagnosis of a solid tumor, such as in the lung. However, Rab27 expression does not correlate with age, sex or tumor size/grade [64]. This correlation between Rab27 expression and patient survival time is linked to the cellular functions of Rab27. Indeed, an increase in Rab27 expression would promote the production of EVs, and therefore the progression of metastases [65]. Rab27b is also involved in the activation of RAS proteins by controlling the MEK/ERK signaling pathway, thereby promoting cell growth. Depletion of Rab27b significantly reduced tumor cell progression in myelomonocytic leukemia [66].

## 4. Conclusions

Initially studied for their involvement in the endosomal maturation pathway, Rab proteins rapidly attracted interest for their role in vesicular transport. With the explosion in the number of studies on EVs over the last two decades, the role of Rab in the biogenesis and secretion of EVs, and more specifically of Rab27 isoforms, has come to light. Taken together, these data make Rab27 a target of interest for controlling cell–cell communication by EVs. In addition, their involvement in pathological mechanisms, such as pigmentation defects or more recently cancerous processes, could also open prospects for studies into therapeutic approaches that target Rab and their effectors. In more general terms, given their roles in various pathophysiological processes, targeting Rab and their effectors to prevent or reinforce certain mechanisms could provide promising opportunities for new targeted therapeutic approaches.

## Figures and Tables

**Table 1:** List of the main Rab involved in vesicular trafficking.

Rab Proteins	Effector(s) and Partners	Role(s) in Vesicular Trafficking
Rab1	p115-GM130	ER-Golgi trafficking
	Giantin-Golgin84	Tethering
Rab2	RUND-1	ER-Golgi trafficking
	CCCP-1	Tethering
Rab4	Rabaptin-4,5,5β	Protein sorting and recycling
	Rabex-5	Endocytic recycling to plasma membrane
Rab5	Rabaptin-5,5β-p150-Vac1-EEA1	Endocytic internalization and early endosome formation
	Rabenosyn-5-Vps34,45-CORVET	
	Rabkinesin-6-Rabex-5-Rabphillin-3	Tethering and fusion
	Syntaxin13,16	
Rab7	Rabring	Late endocytic trafficking
	HOPS complex	Vesicle fusion
Rab8	Rab8IP	Transport between Golgi and TGN
Rab9	p40	Cargo adaptor, sorting and fusion
	TIP47	Exchanges between late endosomes and trans-Golgi
Rab10	MICAL1	Transport between Golgi and TGN
	MYO5A-B-C	Transport between TGN to plasma membrane
	RIMS1	
Rab11	Rabphylin11-Rab11BP	Exocytosis, transport and recycling of endosomes
	FIP2-4-Sec15	Transport from the Golgi
	RIP11-Sec13	Endocytic recycling
Rab14	KIF16B-RUFY1-ZFYVE20	Transport between Golgi and early endosomes
Rab15	REP15	Exit from recycling endosomes
		Inhibitor of endocytin internalization
Rab21	APPL	Endocytic internalization
	ITGA2-ITGA11	Cytokinesis
Rab27a	MLPH-SLP2A-Rabphilin-3	Exocytosis
	Noc2-Granuphilin-CORO1C	
	MYO5A-MYRIP-RPH3A	
	RPH3AL-SYTL1-5-UNC13D	
Rab27b	SYP4-EXPH5	Exocytosis
Rab31	OCRL-TBC1D2B	Bidirectional transport between TGN and early endosomes
Rab35	ACAP2-FSCN1-MICALL1-OCRL	Endocytic recycling
Rab44	Coronin1C	(Putative) Lysosomal function

## Data Availability

Not applicable.

## References

[1] Touchot N., Chardin P., Tavitian A. (1987). Four additional members of the ras gene superfamily isolated by an oligonucleotide strategy: Molecular cloning of YPT-related cDNAs from a rat brain library. Proc. Natl. Acad. Sci. USA.

[2] Pfeffer S.R. (1994). Rab GTPases: Master regulators of membrane trafficking. Curr. Opin. Cell Biol..

[3] Pfeffer S.R. (2007). Unsolved mysteries in membrane traffic. Annu. Rev. Biochem..

[4] Stenmark H. (2009). Rab GTPases as coordinators of vesicle traffic. Nat. Rev. Mol. Cell Biol..

[5] Zerial M., McBride H. (2001). Rab proteins as membrane organizers. Nat. Rev. Mol. Cell Biol..

[6] Pereira-Leal J.B., Seabra M.C. (2000). The mammalian Rab family of small GTPases: Definition of family and subfamily sequence motifs suggests a mechanism for functional specificity in the Ras superfamily. J. Mol. Biol..

[7] Waschbusch D., Khan A.R. (2020). Phosphorylation of Rab GTPases in the regulation of membrane trafficking. Traffic.

[8] Ostermeier C., Brunger A.T. (1999). Structural basis of Rab effector specificity: Crystal structure of the small G protein Rab3A complexed with the effector domain of rabphilin-3A. Cell.

[9] Notredame C., Higgins D.G., Heringa J. (2000). T-Coffee: A novel method for fast and accurate multiple sequence alignment. J. Mol. Biol..

[10] Jumper J., Evans R., Pritzel A., Green T., Figurnov M., Ronneberger O., Tunyasuvunakool K., Bates R., Zidek A., Potapenko A. (2021). Highly accurate protein structure prediction with AlphaFold. Nature.

[11] Varadi M., Anyango S., Deshpande M., Nair S., Natassia C., Yordanova G., Yuan D., Stroe O., Wood G., Laydon A. (2022). AlphaFold Protein Structure Database: Massively expanding the structural coverage of protein-sequence space with high-accuracy models. Nucleic Acids Res..

[12] Wang J., Youkharibache P., Zhang D., Lanczycki C.J., Geer R.C., Madej T., Phan L., Ward M., Lu S., Marchler G.H. (2020). iCn3D, a web-based 3D viewer for sharing 1D/2D/3D representations of biomolecular structures. Bioinformatics.

[13] Ogawa K., Kadowaki T., Tokuhisa M., Yamaguchi Y., Umeda M., Tsukuba T. (2020). Role of the EF-hand and coiled-coil domains of human Rab44 in localisation and organelle formation. Sci. Rep..

[14] Shinde S.R., Maddika S. (2018). Post translational modifications of Rab GTPases. Small GTPases.

[15] Lai Y.C., Kondapalli C., Lehneck R., Procter J.B., Dill B.D., Woodroof H.I., Gourlay R., Peggie M., Macartney T.J., Corti O. (2015). Phosphoproteomic screening identifies Rab GTPases as novel downstream targets of PINK1. EMBO J..

[16] Steger M., Tonelli F., Ito G., Davies P., Trost M., Vetter M., Wachter S., Lorentzen E., Duddy G., Wilson S. (2016). Phosphoproteomics reveals that Parkinson’s disease kinase LRRK2 regulates a subset of Rab GTPases. Elife.

[17] van der Sluijs P., Hull M., Webster P., Male P., Goud B., Mellman I. (1992). The small GTP-binding protein rab4 controls an early sorting event on the endocytic pathway. Cell.

[18] Fitzgerald M.L., Reed G.L. (1999). Rab6 is phosphorylated in thrombin-activated platelets by a protein kinase C-dependent mechanism: Effects on GTP/GDP binding and cellular distribution. Biochem. J..

[19] Hutagalung A.H., Novick P.J. (2011). Role of Rab GTPases in membrane traffic and cell physiology. Physiol. Rev..

[20] Jin H., Tang Y., Yang L., Peng X., Li B., Fan Q., Wei S., Yang S., Li X., Wu B. (2021). Rab GTPases: Central Coordinators of Membrane Trafficking in Cancer. Front. Cell Dev. Biol..

[21] van der Sluijs P., Hull M., Huber L.A., Male P., Goud B., Mellman I. (1992). Reversible phosphorylation–dephosphorylation determines the localization of rab4 during the cell cycle. EMBO J..

[22] Candela P., Gosselet F., Miller F., Buee-Scherrer V., Torpier G., Cecchelli R., Fenart L. (2008). Physiological pathway for low-density lipoproteins across the blood-brain barrier: Transcytosis through brain capillary endothelial cells in vitro. Endothelium.

[23] Harding C., Heuser J., Stahl P. (1983). Receptor-mediated endocytosis of transferrin and recycling of the transferrin receptor in rat reticulocytes. J. Cell Biol..

[24] Pan B.T., Johnstone R.M. (1983). Fate of the transferrin receptor during maturation of sheep reticulocytes in vitro: Selective externalization of the receptor. Cell.

[25] Gibieza P., Petrikaite V. (2021). The dual functions of Rab11 and Rab35 GTPases-regulation of cell division and promotion of tumorigenicity. Am. J. Cancer Res..

[26] Singh R.D., Puri V., Valiyaveettil J.T., Marks D.L., Bittman R., Pagano R.E. (2003). Selective caveolin-1-dependent endocytosis of glycosphingolipids. Mol. Biol. Cell.

[27] Shikanai M., Ito S., Nishimura Y.V., Akagawa R., Fukuda M., Yuzaki M., Nabeshima Y.I., Kawauchi T. (2023). Rab21 regulates caveolin-1-mediated endocytic trafficking to promote immature neurite pruning. EMBO Rep..

[28] Bielli A., Thornqvist P.O., Hendrick A.G., Finn R., Fitzgerald K., McCaffrey M.W. (2001). The small GTPase Rab4A interacts with the central region of cytoplasmic dynein light intermediate chain-1. Biochem. Biophys. Res. Commun..

[29] Cormont M., Meton I., Mari M., Monzo P., Keslair F., Gaskin C., McGraw T.E., Le Marchand-Brustel Y. (2003). CD2AP/CMS regulates endosome morphology and traffic to the degradative pathway through its interaction with Rab4 and c-Cbl. Traffic.

[30] de Renzis S., Sonnichsen B., Zerial M. (2002). Divalent Rab effectors regulate the sub-compartmental organization and sorting of early endosomes. Nat. Cell Biol..

[31] Strick D.J., Elferink L.A. (2005). Rab15 effector protein: A novel protein for receptor recycling from the endocytic recycling compartment. Mol. Biol. Cell.

[32] Ullrich O., Reinsch S., Urbe S., Zerial M., Parton R.G. (1996). Rab11 regulates recycling through the pericentriolar recycling endosome. J. Cell Biol..

[33] Hales C.M., Vaerman J.P., Goldenring J.R. (2002). Rab11 family interacting protein 2 associates with Myosin Vb and regulates plasma membrane recycling. J. Biol. Chem..

[34] Kouranti I., Sachse M., Arouche N., Goud B., Echard A. (2006). Rab35 regulates an endocytic recycling pathway essential for the terminal steps of cytokinesis. Curr. Biol..

[35] Rink J., Ghigo E., Kalaidzidis Y., Zerial M. (2005). Rab conversion as a mechanism of progression from early to late endosomes. Cell.

[36] Gilleron J., Zeigerer A., Marsico G., Galvez T., Zerial M. (2012). Key role of Rab5: From endosome biogenesis to liver metabolism. Med. Sci..

[37] Dong B., Kakihara K., Otani T., Wada H., Hayashi S. (2013). Rab9 and retromer regulate retrograde trafficking of luminal protein required for epithelial tube length control. Nat. Commun..

[38] Ganley I.G., Carroll K., Bittova L., Pfeffer S. (2004). Rab9 GTPase regulates late endosome size and requires effector interaction for its stability. Mol. Biol. Cell.

[39] Guerra F., Bucci C. (2016). Multiple Roles of the Small GTPase Rab7. Cells.

[40] Luzio J.P., Pryor P.R., Bright N.A. (2007). Lysosomes: Fusion and function. Nat. Rev. Mol. Cell Biol..

[41] Kuchitsu Y., Homma Y., Fujita N., Fukuda M. (2018). Rab7 knockout unveils regulated autolysosome maturation induced by glutamine starvation. J. Cell Sci..

[42] Kadowaki T., Yamaguchi Y., Ogawa K., Tokuhisa M., Okamoto K., Tsukuba T. (2021). Rab44 isoforms similarly promote lysosomal exocytosis, but exhibit differential localization in mast cells. FEBS Open Bio..

[43] Wei D., Zhan W., Gao Y., Huang L., Gong R., Wang W., Zhang R., Wu Y., Gao S., Kang T. (2021). RAB31 marks and controls an ESCRT-independent exosome pathway. Cell Res..

[44] van Niel G., Carter D.R.F., Clayton A., Lambert D.W., Raposo G., Vader P. (2022). Challenges and directions in studying cell-cell communication by extracellular vesicles. Nat. Rev. Mol. Cell Biol..

[45] van Niel G., D’Angelo G., Raposo G. (2018). Shedding light on the cell biology of extracellular vesicles. Nat. Rev. Mol. Cell Biol..

[46] Buzas E.I. (2023). The roles of extracellular vesicles in the immune system. Nat. Rev. Immunol..

[47] Saint-Pol J., Gosselet F. (2018). Small but sturdy: Neuronal-derived exosomes control brain vasculature integrity. Med. Sci..

[48] Saint-Pol J., Gosselet F., Duban-Deweer S., Pottiez G., Karamanos Y. (2020). Targeting and Crossing the Blood-Brain Barrier with Extracellular Vesicles. Cells.

[49] Ostrowski M., Carmo N.B., Krumeich S., Fanget I., Raposo G., Savina A., Moita C.F., Schauer K., Hume A.N., Freitas R.P. (2010). Rab27a and Rab27b control different steps of the exosome secretion pathway. Nat. Cell Biol..

[50] Jongsma M.L., Bakker J., Cabukusta B., Liv N., van Elsland D., Fermie J., Akkermans J.L., Kuijl C., van der Zanden S.Y., Janssen L. (2020). SKIP-HOPS recruits TBC1D15 for a Rab7-to-Arl8b identity switch to control late endosome transport. EMBO J..

[51] Hsu C., Morohashi Y., Yoshimura S., Manrique-Hoyos N., Jung S., Lauterbach M.A., Bakhti M., Gronborg M., Mobius W., Rhee J. (2010). Regulation of exosome secretion by Rab35 and its GTPase-activating proteins TBC1D10A-C. J. Cell Biol..

[52] Savina A., Fader C.M., Damiani M.T., Colombo M.I. (2005). Rab11 promotes docking and fusion of multivesicular bodies in a calcium-dependent manner. Traffic.

[53] Savina A., Vidal M., Colombo M.I. (2002). The exosome pathway in K562 cells is regulated by Rab11. J. Cell Sci..

[54] Yang L., Peng X., Li Y., Zhang X., Ma Y., Wu C., Fan Q., Wei S., Li H., Liu J. (2019). Long non-coding RNA HOTAIR promotes exosome secretion by regulating RAB35 and SNAP23 in hepatocellular carcinoma. Mol. Cancer.

[55] Verweij F.J., Bebelman M.P., George A.E., Couty M., Becot A., Palmulli R., Heiligenstein X., Sires-Campos J., Raposo G., Pegtel D.M. (2022). ER membrane contact sites support endosomal small GTPase conversion for exosome secretion. J. Cell Biol..

[56] Fukuda M. (2006). Rab27 and its effectors in secretory granule exocytosis: A novel docking machinery composed of a Rab27.effector complex. Biochem. Soc. Trans..

[57] Tsuboi T., Fukuda M. (2006). Rab3A and Rab27A cooperatively regulate the docking step of dense-core vesicle exocytosis in PC12 cells. J. Cell Sci..

[58] Jeppesen D.K., Fenix A.M., Franklin J.L., Higginbotham J.N., Zhang Q., Zimmerman L.J., Liebler D.C., Ping J., Liu Q., Evans R. (2019). Reassessment of Exosome Composition. Cell.

[59] Shen Y.T., Gu Y., Su W.F., Zhong J.F., Jin Z.H., Gu X.S., Chen G. (2016). Rab27b is Involved in Lysosomal Exocytosis and Proteolipid Protein Trafficking in Oligodendrocytes. Neurosci. Bull..

[60] Uhlen M., Fagerberg L., Hallstrom B.M., Lindskog C., Oksvold P., Mardinoglu A., Sivertsson A., Kampf C., Sjostedt E., Asplund A. (2015). Proteomics. Tissue-based map of the human proteome. Science.

[61] Fukuda M. (2021). Rab GTPases: Key players in melanosome biogenesis, transport, and transfer. Pigment Cell Melanoma Res..

[62] Barral D.C., Ramalho J.S., Anders R., Hume A.N., Knapton H.J., Tolmachova T., Collinson L.M., Goulding D., Authi K.S., Seabra M.C. (2002). Functional redundancy of Rab27 proteins and the pathogenesis of Griscelli syndrome. J. Clin. Investig..

[63] Izumi T. (2021). In vivo Roles of Rab27 and Its Effectors in Exocytosis. Cell Struct. Funct..

[64] Koh H.M., Jang B.G., Kim D.C. (2020). Prognostic significance of Rab27 expression in solid cancer: A systematic review and meta-analysis. Sci. Rep..

[65] Li J., Jin Q., Huang F., Tang Z., Huang J. (2017). Effects of Rab27A and Rab27B on Invasion, Proliferation, Apoptosis, and Chemoresistance in Human Pancreatic Cancer Cells. Pancreas.

[66] Ren J.G., Xing B., Lv K., O’Keefe R.A., Wu M., Wang R., Bauer K.M., Ghazaryan A., Burslem G.M., Zhang J. (2023). RAB27B controls palmitoylation-dependent NRAS trafficking and signaling in myeloid leukemia. J. Clin. Investig..

